# Effect of *Juniperus communis* extract on probiotic properties of *Bacillus safensis* isolated from camel milk in the region of El Oued (Algeria)

**DOI:** 10.1002/fsn3.4262

**Published:** 2024-06-20

**Authors:** Ikram Layadi, Ammar Touhami Laiche, Mohammed Laid Tlili, Mohammed Messaoudi, Djilani Ghemam Amara, Maha Mezghani‐Khemakhem, Chahnez Naccache, Barbara Sawicka, Maria Atanassova, Wafa Zahnit, Sheikh F. Ahmad

**Affiliations:** ^1^ Laboratory of Biodiversity and Application of Biotechnology in the Agricultural Field, Faculty of the Sciences of Nature and Life University of El Oued El Oued Algeria; ^2^ Department of Biology, Faculty of Sciences of Nature and Life University of El‐Oued El Oued Algeria; ^3^ Laboratory of Biogeochemistry of Desert Environments Laboratory University of Ouargla Ouargla Algeria; ^4^ Department of Cellular and Molecular Biology, Faculty of Sciences of Nature and Life University of El‐Oued El Oued Algeria; ^5^ Nuclear Research Centre of Birine Djelfa Algeria; ^6^ Laboratory of Biology, Environment and Health, Department of Biology, Faculty of Life and Natural Sciences University of El Oued El Oued Algeria; ^7^ Laboratory of Biochemistry and Biotechnology (LR01ES05), Department of Biology, Faculty of Sciences of Tunis University of Tunis El Manar Tunis Tunisia; ^8^ Department of Plant Production Technology and Commodities Science University of Life Sciences in Lublin Lublin Poland; ^9^ Scientific Consulting, Chemical Engineering University of Chemical Technology and Metallurgy Sofia Bulgaria; ^10^ Laboratory of Valorization and Promotion of Saharan Resource (VPRS), Faculty of Mathematics and Matter Sciences University of Ouargla Ouargla Algeria; ^11^ Department of Pharmacology and Toxicology, College of Pharmacy King Saud University Riyadh Saudi Arabia

**Keywords:** *Bacillus safensis*, camel milk, *Juniperus communis*, polyphenols, probiotics

## Abstract

The current study focuses on the effect of *Juniperus communis* extract on the probiotic properties of lactic acid bacteria isolated from camel milk in the region of El Oued (Algeria). Chromatographic analysis by HPLC was carried out to detect the most important compounds extracted from the plant. The total phenolic and flavonoid contents were determined using the colorimetric procedures Folin–Ciocalteu and aluminum chloride. The probiotic properties were studied and evaluated in vivo with *Juniperus communis* extracts after isolating strains from camel's milk and identifying them using 16S rRNA gene sequencing. Chromatographic profiles of the phenolic compounds revealed that *Juniperus communis* extract is rich in quercetin. After conducting chemical analyses of polyphenols and flavonoids, the results demonstrated a high content of phenolic compounds in *Juniperus communis* extracts (polyphenols: 103.80 ± 0.30 mg GAE/g E. flavonoids: 15.85 ± 0.80 mg QE/g E). Sequencing and phylogenetic analysis showed that the isolates belong to *Bacillus pumilus* and *Bacillus safensis* strains. The combination of *Juniperus communis* and *Bacillus safensis* restored the healthy intestine wall structure and returned the blood biochemical parameters to normal values. It was found that the mixture enhanced anti‐inflammatory effectiveness by reducing erythrocyte sedimentation rate and C‐reactive protein values. *Juniperus communis* has a high polyphenol and flavonoid content which can have a considerable impact on *Bacillus safensis* probiotic properties.

## Graphical abstract

Current study focuses on the effect of *Juniperus communis* extract on the probiotic properties of lactic acid bacteria isolated from camel milk. Chromatographic profiles of the phenolic compounds revealed that *Juniperus communis* extract is rich in quercetin. The results demonstrated a high content of phenolic compounds in *Juniperus communis* extracts (polyphenols: 103.80 ± 0.30 mg GAE/g E. flavonoids: 15.85 ± 0.80 mg QE/g E). *Juniperus communis* has a high polyphenol and flavonoid content which can have a considerable impact on *Bacillus safensis* probiotic properties.

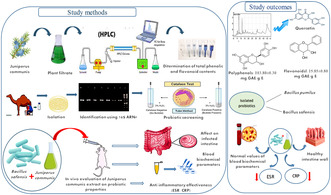

## 1 INTRODUCTION

According to FAO and WHO, probiotics are “live microorganisms that when administered in adequate amounts, confer health benefits on the host.” Probiotics, which may exist in the intestines and foods and supplements, are a significant class of beneficial bacteria that are ingested or supplemented nowadays (Hill et al., 2014). Probiotic use has been shown to improve immune functioning by interacting with various immune cells and altering the makeup of the intestinal microbiota. As a result, probiotics' ability to promote health and modulate immunity is generally recognized (Mazziotta et al., 2023). These bacteria were generated differently depending on the probiotics used. The human large and small intestines, breast milk, animal origins, and food sources like raw or fermented milk are examples of biological sources (Shewale et al., 2014). Probiotics can be found mainly in dairy products, and they secrete their enzymes and substances of interest; the quality and safety of dairy products are important guarantees for the revitalization of the dairy industry. Among the many issues related to dairy safety, bioactive components have attracted the attention of government, researchers, and citizens due to their high importance (Xiong, Chen, et al., 2022; Xiong, Wen, et al., 2022). Likewise, whey fermentation combined with probiotics has been widely used in the development of hypoallergenic milk in recent years (Zhao et al., 2023). Since plant foods are high in antioxidants, which are crucial for preventing disease, they are often a good source with a range of therapeutic effects. The idea of incorporating probiotics into a plant matrix is still being considered a critical component in order to enhance the activity of probiotic strains and protect these bacteria from the deleterious effects of oxygen and its active derivatives (Spacova et al., 2020).

Numerous studies have been conducted on the effects of plant extracts rich in polyphenolic compounds on the growth of probiotic bacteria and other microorganisms. Plant extracts have been shown to suppress the development of bacteria connected to food degradation, as well as pathogenic and physiological microflora intestinal issues (Haddadin, 2010). *Juniperus communis* has been used in traditional medicine since ancient times and is recognized as an important medicinal plant (Dhaka & Mittal, 2021), which includes treating tumors, bronchitis, indigestion, diarrhea, and stomach pain (Mansouri et al., 2011), This is because it contains a sizable amount of phenolic chemicals, which have been demonstrated to have beneficial effects on a range of biological systems (Dhaka & Mittal, 2021). Additionally, these chemicals have the ability to modify the microbiota in the intestine by selectively promoting the growth of lactobacilli and bifidobacteria while inhibiting the growth of pathogenic bacteria like Clostridia (Tuck & Hayball, 2002).

This research's original concept was to isolate and purify lactic acid bacteria from camel milk, which possesses probiotic qualities. Also to investigate how *Juniperus communis* extract influences these strains' probiotic qualities.

## 2 MATERIALS AND METHODS

### 2.1 Biological material

*Juniperus communis* was collected on September 27, 2022, from Wanza region (Tebessa, Algerian east, 35° 57′ 00″ N, 8° 08′ 00″ E). The aerial parts were washed with water, dried, and ground. Then it was stored at ambient temperature in a glass box in a dry and protected place until use. A milk sample was collected from camel (*Camelus dromedarius*) from Al‐Nakhla area (Al‐Wed) during January 2023, which was 11 years old and fed on son (Bran) and olive pomace (Fatura).

### 2.2 Extract preparation

The extraction was carried out according to the methods of Biswas et al. (2017) with some modifications. 10 g of the sample was put in contact with 200 mL of distilled water in a glass Erlenmeyer flask at room temperature for 24 h. The aqueous extract was recovered after filtration using N°01 filter paper. Distilled water was removed from the filtrate by evaporation under reduced pressure in a rotary evaporator, then oven for at least 48 h at a temperature not exceeding 40°C, and stored until use.

### 2.3 Chromatographic analysis by (HPLC)

Shim‐pack VP‐ODS C18 (4.6 mm × 250 mm, 5 μm), a Shimadzu type analytical column was utilized in a high‐performance liquid chromatography (HPLC) system, which was equipped with a universal injector (Hamilton 25 μL). It was a Shimadzu UV–VIS detector SPD 20A (Thammana, 2016).

The analysis was carried out according to the method described by Jun et al. (2014). A solution of plant extract in the volume of 20 μL was injected into the mobile phase flow. Detection at = 268 nm was used to identify the separated chemicals using the column for 40–50 min.

### 2.4 Qualitative phytochemical analysis

Based on color and/or precipitation reactions, a phytochemical examination is required to pinpoint the major families of secondary metabolites found in the studied plant's leaves, including polyphenols, tannins, alkaloids, saponins, flavonoids, terpenoids, free quinones, and reducing sugars. based on the experimental guidelines of Evans (2009).

### 2.5 Quantitative phytochemical analysis

#### 2.5.1 Determination of total phenolic content

200 μL of the extract are combined with 1 mL of newly made Folin–Ciocalteu reagent (10 times diluted) and 0.8 mL of 7.5% sodium carbonate (Na_2_CO_3_). Using a spectrophotometer set to 765 nm, the whole sample is read against a blank after being incubated at room temperature for 30 min. From the range of the linear calibration curve made with gallic acid, polyphenol concentrations were calculated. The outcomes are given as milligram equivalents of gallic acid (mg GAE/g E) per gram of dry extract (Wong et al., 2006).

#### 2.5.2 Determination of total flavonoid content

The AlCl_3_ solution (2% in methanol) was combined with 1 mL of sample (made in ethanol or in distilled water). Using a spectrophotometer set to 430 nm, the absorbance was measured after 10 min of incubation. According to the curve quercetin calibration, the findings are given in mg equivalent quercetin per gram of dry plant material (Bahorun et al., 1996).

### 2.6 Isolation and identification of probiotics strains

#### 2.6.1 Isolation of probiotic strains

In sterile tubes with a capacity of 10 mL each, the milk samples were dispersed. One milliliter of the parent suspension was used to dilute the milk in decimal amounts (10^−1^, 10^−2^, and 10^−3^) in a physiological saline solution. Into the mass of M17 agar, 1 mL of each dilution was inoculated, and the plates were then incubated in the oven at 37 and 45°C (Ayyash et al., 2018). In order to maintain the purity of the cultures after isolation, colonies were subcultured on M17 medium and incubated at 30 or 45°C. The streak technique was used to purify strains on agar medium, and this was followed by microscopic examination (Laiche et al., 2019).

#### 2.6.2 Phenotypic and molecular identification of probiotics strains

Microscopic and microscopic examinations were performed followed by biochemical and physiological tests including the catalase test, API 10s exhibit, growth at different temperatures (10 and 45°C), and NaCl sensitivity (2%, 4%, and 6.5%).

The NucleoSpin Soil Kit (Macherey‐Nagel, USA) was used to extract the genomic DNA from the bacterial isolates in accordance with the manufacturer's instructions. Thermo Scientific™, USA provided the Nanodrop spectrophotometer, which was used to measure the amount and purity of the DNA extracts. Using the universal bacterial primers for 16S rRNA 27F (5′‐ AGAGTTTGATCMTGGCTCAG‐3′) and 1492R (5′‐GGTTACCTTGTTACGACTT‐3′), a 1500‐bp fragment of the 16S rRNA gene was amplified by PCR (Jansen et al., 2006). The thermal cycling conditions were as follows: a 4‐min initial denaturation at 96°C, 35 cycles of 30 s at 94°C, 30 s at 57°C, and 30 s at 72°C, and a 5‐min final extension at 72°C. The PCR products were processed for 45 min at 100 V in a gel electrophoresis system using 1% agarose gel 1xTE buffer. The Wizard SV Gel and PCR Clean‐up System (Promega, New England) was utilized to purify the 16S rRNA PCR products, and an ABI‐PRISM 3700 DNA automated sequencer (Applied Biosystems) was utilized for sequencing. The sequences were first edited using 4peaks V1.8 to ascertain their identity. They were then submitted to BLASTN (National Center for Biotechnology Information; http://blast.ncbi.nlm.nih.gov/Blast.cgi) for comparison with sequences that had been published on GenBank based on identity ranking (>97%) and E‐values (0.0) (Nguyen et al., 2016).

### 2.7 Selection of probiotic strains

#### 2.7.1 Acidity tolerance

The resistance of bacteria to acidic pH was determined according to the method described by Das et al. (2016) with some modifications. Bacterial strains were activated at 37°C for 16–18 h in a volume of 4 mL of sterile nutritional broth. The bacterial cultures were then centrifuged at 2500 for 10 min before being cleaned with PBS solution. The recycled pellets were centrifuged at 1000 rpm for 5 min, and then washed with PBS buffer. The pellet was divided into two tubes each containing 4 mL of feed broth, then HCl and NaOH were used to bring the pH value of each tube to 2 or 3. Successive dilutions were made up to 10^−2^ after exposure to acidic pH at *t* = 0 and *t* = 3 h. After inoculating the mass onto nutrient agar, these dilutions were incubated at 37°C for 24–48 h. At *t* = 0 and *t* = 3, the viable count was calculated. The quantity of live cells is then measured.

#### 2.7.2 Bile tolerance

One of the essential probiotic selection characteristics is a strain's capacity to tolerate conditions comparable to those found in the human small intestine (Ren et al., 2014). In 4 mL of sterile nutritional broth, bacteria were activated at 37°C for 16–18 h. The pellets were then centrifuged once more for 5 min at a speed of 1000 rpm, followed by a PBS buffer wash. Divide the granules into three tubes, each of which contains 3 mL of nutritional broth and 1%, 3%, or 5% bile, respectively. Following a bulk inoculation on nutrient agar, they were incubated for 24–48 h at 37°C. Decide the relevant number at *t* = 0 h and at *t* = 3 h.

#### 2.7.3 Antimicrobial activity

Using the Barefoot and Klaenhammer (1984) well diffusion method, sterile Petri dishes were filled with a volume of Mueller‐Hinton agar media. After the medium has solidified, the pathogenic strain suspension is injected onto the plates, and then sterile 6‐mm‐diameter wells are excavated using a cookie cutter. (Durham Bell) on the M17 agar, which will be filled with 60–80 μL of filtered and neutralized supernatant after a lactic strain culture was centrifuged at 4000 rpm for 15 min. Zones of inhibition surrounding the wells indicate antibacterial activity after the dishes have been incubated at 37°C for 24 h.

#### 2.7.4 Antibiotic sensitivity

This test was performed using the method of Bazireh et al. (2020) but with some minor changes. On M17 agar medium, the lactic strains were grown and kept at 30°C for 24 h. Each strain's bacterial colonies were then put into physiological water after having their DO adjusted to 0.5 McFarland. The antibiotic discs, which were comprised of gentamycin (120 g), penicillin (10 g), oxacilline (5 g), amoxicillin (25 g), and ciproflaxacine (5 g), were then put on the surface (four per box) of the dishes containing M17 agar. The zones of inhibition were determined by including the antibiotic disk's (6 mm) diameter in the zone's breadth after the dishes had been incubated at 30°C for 48 h.

### 2.8 In vivo evaluation of *Juniperus communis* extract on probiotic properties

#### 2.8.1 Induction of intestinal inflammation

The rats were divided into 4 groups of 5 individuals each: The first group received distilled water by gavage (control group). The second group received a dose of 0.5 mL of castor oil after fasting for 16 h every day by gavage, for a week (infected group). The third group received a treatment of 0.5 mL of a probiotic lactic acid bacteria (*Bacillus safensis*) diluted in 5 mL of physiological water, for 1 week. The fourth group was treated daily with a mixture of *Bacillus safensis* solution and 200 mg/kg of *Juniperus communis* aqueous extract (JAE).

#### 2.8.2 Blood samples

Blood sampling is done at the time of sacrifice of the rats (the rats were fasted for 24 h before being sacrificed), and the blood collected in EDTA tubes to be used for the biochemical parameter assays (Blood count: BCN). Then the samples were centrifuged for 15 min at 4000 rpm. The plasma was collected and frozen until it was used for the determination of certain inflammatory assessment parameters: C‐reactive protein (CRP) and erythrocyte sedimentation rate (ESR).

#### 2.8.3 Histological study

After washing the organs with physiological saline solution (NaCl 0.9%), the intestine was fixed in alcoholic Bouin. The sections were frozen after the incisions were made using a Leica microtome to a thickness of 6 μm. The hematoxylin‐eosin stain was used on the cuts, with the hematoxylin giving the nucleus a blue‐purple color. Conversely, eosin is causing the cytoplasm and other fundamental cellular components to become pink (Tlili et al., 2017).

## 3 RESULTS

### 3.1 Chromatographic analysis (HPLC)

**FIGURE 1:**
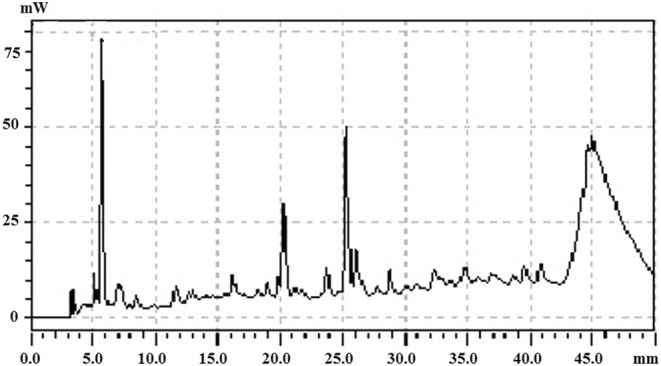
HPLC chromatographic profile of *Juniperus communis* aqueous extract.

The chromatogram (Figure 1) shows the results of the chromatographic analysis. HPLC detected seven phenolic components in the aqueous extract of *Juniperus communis*. The concentration of these compounds and their retention period are presented in Table 1.

**TABLE 1:** Some phenolic compounds identified by HPLC in aqueous extract of *Juniperus communis*.

Aqueous extract of *Juniperus communis*		
Retention time (min)	Compounds	Concentration (μg/mg extract)
5.296	Gallic acid	0.827
13.610	Chlorogenic acid	1.270
15.538	Vanilic acid	0.294
16.244	Caffeic acid	1.642
28.175	Rutin	0.375
34.897	Naringin	1.331
44.897	Quercetin	8.794

### 3.2 Qualitative phytochemical analysis

A phytochemical screening was performed (Table 2) to determine whether any chemical groups were present in *Juniperus communis* extract.

**TABLE 2:** Phytochemical qualitative analysis of *Juniperus communis* aqueous extract.

Active constituents	*Juniperus communis* aqueous extract
Polyphenols	+
Tannins	+
Flavonoids	+
Alkaloids	+
Terpenoids	+
Saponins	−
Free quinones	+
Reducing sugars	+
Cardiac glycosides	+

*Note*: +: Present compound; −: Absent compound.

From these results, it is found that most of the components present in the extract are: polyphenols, tannins, terpenoids, and free quinones in large quantities, but the absence of alkaloids is observed.

### 3.3 Quantitative phytochemical analysis

According to the results, it was found that the extract has a high total polyphenol content, with a value of 103.80 ± 0.30 mg GAE/g E. Regarding the quantitative total flavonoid content, *Juniperus communis* extract is rich in flavonoids (15.85 ± 0.80 mg QE/g E).

### 3.4 Isolation and identification of probiotic strains

#### 3.4.1 Isolation of probiotic strains

Based on biochemical tests and morphological characteristics, eight colonies were isolated after gram staining, but we chose only four to study their probiotic properties. They are symbolized by SM45°C, 10^−1^ 45°C, 10^−1^ 37°C, and 10^−2^ 37°C.

#### 3.4.2 Phenotypic and molecular identification of probiotic strains

**FIGURE 2:**
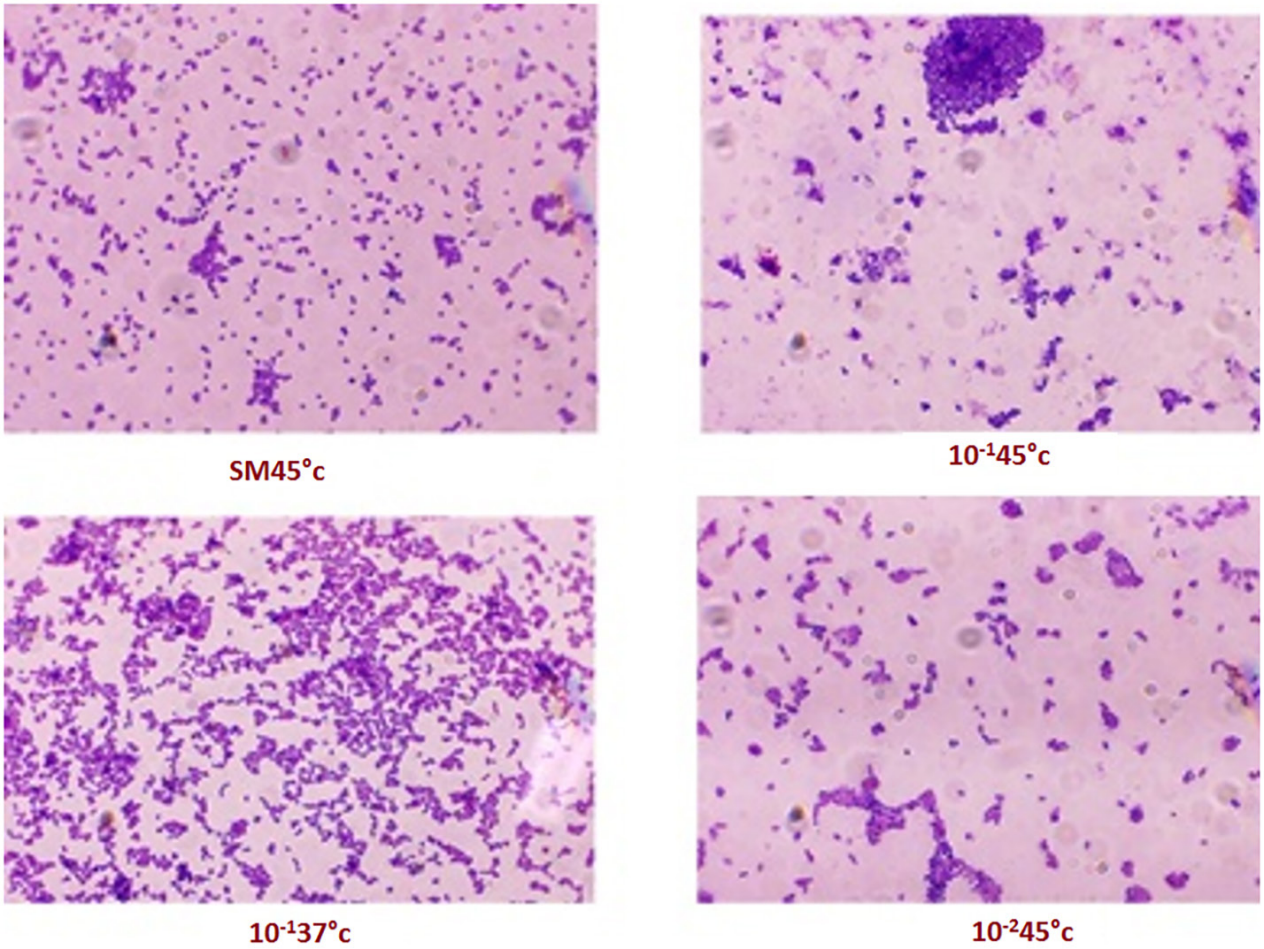
Colonies' microscopic appearance (Gx100).

From a macroscopic examination of the cultures on M17 agar, the isolates were both big and small, white, and creamy. The homogeneous form of bacteria reflects their purity, this indicates that every strain had a Gram‐positive. Microscopic observation revealed that the form of cells is ovoid or spherical cocci form, arranged in pairs, or arranged in chains (Figure 2).

The strains are generally catalase (+)‐positive and showed better growth at different concentrations of NaCl (2%, 4%, and 6.5%). Probiotic strains demonstrated similarity in growth at the incubation temperature of 10°C, a significant increase in growth is observed for most of the strains at 45°C. Based on macroscopic, microscopic aspects, as well as on certain biochemical and physiological tests (Table 3), 50% of the isolates were identified as *Streptococcus thermophiles* and these included SM45°C, 10^−1^ 45°C. Fifty percent of the isolates belonged to *Lactococcus lactis* (10^−1^ 37°C, 10^−2^ 37°C).

**TABLE 3:** Biochemical and physiological criteria of probiotics isolated from camel milk.

	Tests		Strains			
			SM45°C	10^−1^ 45°C	10^−1^ 37°C	10^−2^ 37°C
Api 10 s Gallery	Catalase		+	+	+	+
	T	45°C→10°C	−	−	/	/
		37°C→10°C	/	/	++	++
		37°C→45°C	/	/	+++	++
	NaCl	2%	+++	+	++++	++++
		4%	++	++	++++	++++
		6.5%	+++	++	++++	++++
	PH		+++	+++	/	/
	ONPG		+	+	+	+
	GLU		+	+	+	+
	ARA		+	+	+	+
	LDC		+	+	+	+
	ODC		+	+	+	+
	CIT		+	+	+	+
	H_2_ S		+	+	+	+
	URE		−	−	−	−
	TDA		+	+	+	+
	IND		+	+	+	−

Abbreviations: ARA, L‐arabinose resistance test; CIT, Citrate test; GLU, Glucose test; H2S, Hydrogen Sulfide test; IND, Indol test; LDC, Lysine decarboxylase test; NaCl, Sodium chloride; ODC, Ornithine decarboxylase test; ONPG, O‐Nitrophenyl‐β‐D‐Galactopyranoside test; Ph, Potential of hydrogen; TDA, Tryptophan‐deaminase activity; URA, Urease test; T, Temperature.

The DNA sequencing results were analyzed using NCBI‐BLAST. The analysis revealed that the 16S rDNA sequence of *Streptococcus thermophilus* exhibited the highest degree of identity with *Bacillus pumilus* (accession number: NZ_PTXV01000013.1, 99%), while *Lactococcus lactis* showed identity with *Bacillus safensis* (accession number: NZ_CP043404.1, 100%).

#### 3.4.3 Selection of probiotic strains

##### Acidity tolerance

The growth of *Bacillus safensis* decreased after incubating at pH = 2 conversely at pH = 3. it increased and reached its greatest value. The growth of *Bacillus pumilus* is increased in all pH values after incubation. The highest values were after incubation at pH = 3 (Table 4).

**TABLE 4:** Effect of acidic pH on *Bacillus pumilus* and *Bacillus safensis* viability in (log CFU/mL).

Strains	pH = 2		pH = 3	
*Bacillus pumilus*	*t* = 0 h	*t* = 3 h	*t* = 0 h	*t* = 3 h
10^−1^	2.710	2.173	2.767	5.136
10^−2^	3.158	3.822	3.843	3.915
	2.934 ± 0.316	2.997 ± 1.166	3.305 ± 0.760	4.525 ± 0.863
*Bacillus safensis*	*t* = 0 h	*t* = 3 h	*t* = 0 h	*t* = 3 h
10^−1^	1.806	1.643	2.675	2.853
10^−2^	2.643	2.799	3.694	3.880
	2.224 ± 0.591	2.221 ± 0.817	3.184 ± 0.720	3.366 ± 0.726

##### Bile tolerance

The findings, which are presented in Table 5, indicate that all strains are tolerant to bile salts at concentrations of 1%, 3%, and 5%, with the majority exhibiting better survival rates at the latter.

**TABLE 5:** Effect of bile salts on *Bacillus pumilus and Bacillus safensis* viability in (log CFU/mL).

Strains	Concentration de bile %					
	1%	1%	3%		5%	
	*t*:0 h	*t*:3 h	*t*:0 h	*t*:3 h	*t*:0 h	*t*:3 h
*Bacillus pumilus*	1.363	1.416	1.912	1.567	1.547	1.625
*Bacillus safensis*	1.852	1.713	1.404	1.660	1.519	1.641

### 3.5 Antimicrobial activity

According to the results, the strains of probiotics selected do not represent any antimicrobial activity against *Enterococcus faecalis*, *Staphylococcus haemolyticus*, *Staphylococcus aureus*, *Candida albicans*, *Klebsiella* sp., *Salmonella* sp., and *Escherichia coli*.

#### 3.5.1 Antibiotic sensitivity

According to Table 6, it can be concluded that *Bacillus safensis* is highly resistant to oxacillin, penicillin, and amoxicillin; however, it is susceptible to gentamicin and ciproflaxacone. While *Bacillus pumilus* was susceptible to gentamicin, ciproflaxacone, and amoxicillin, it was resistant to oxacillin and penicillin.

**TABLE 6:** Antibiotic test results of *Bacillus pumilus* and *Bacillus safensis*.

Antibiotics	*Bacillus pumilus*	*Bacillus safensis*
GEN 10	S (12 mm)	S (15 mm)
AX 10	S (24 mm)	R (6 mm)
CIP 5	S (18 mm)	S (24 mm)
OX10	R (6 mm)	R (6 mm)
P10	R (6 mm)	R (6 mm)

Abbreviations: R, Resistant; S, Susceptible.

### 3.6 In vivo evaluation of *Juniperus communis* extract on probiotic properties

#### 3.6.1 Macroscopic observation

The results of the macroscopic study (Table 7) showed that group II rats which did not receive any treatment, had signs of infection accompanied by diarrhea compared with control rats. These symptoms disappeared (group III and IV) after the administration of treatment solutions. Macroscopic observation provisionally confirms the protective effect of the studied plant with *Bacillus safensis* strains.

**TABLE 7:** Macroscopic results of experimental animals during in vivo study.

In infection period	Group I (Control)	Group II (Infected)	Group III (*Bacillus safensis* Probiotic treatment)	Group IV (*Bacillus safensis +* JAE treatment)
Diarrhea indicator	−	++	−	+
Body weight (g)				
Before	220.4 ± 4.56	203.2 ± 19.11	210.2 ± 23.76	211.2 ± 15.35
After	224 ± 4.63	188.4 ± 13.31	206.2 ± 23.88	203.8 ± 13.17
After dissection				
Intestine color	Normal	Dark brown	Brown	Normal
Intestine shape	Normal	Irritated	Normal	Normal
Intestine diameter(cm)	0.3–0.4	0.2–0.3	0.2–0.4	0.3–0.5

#### 3.6.2 Microscopic observation

Microscopic observation of the histological section of the intestine in the control group shows a healthy structure accompanied by a healthy intestinal wall composed of the mucosa (epithelium, lamina propria, muscular mucosa), the submucosa, the muscularis, and the sub‐serous, with healthy and normal villi also a total absence of inflammation and no tissue/cellular damage, with an absence of necrosis. Therefore, the histological section shows a more or less regular tissue appearance. Regarding the rats that received castor oil, after dissection, microscopic observation of the histological sections revealed a destroyed structure of intestinal cells plus the presence of signs of very serious infection represented by inflammation that caused the grouping of lymphocytic cells, a reduction in the height of the villi, and some villi appearing necrotic, plus the presence of cellular lesions, it was observed that the lamina propria was completely destroyed, these observations revealed that these rats suffered from diarrhea.

**FIGURE 3:**
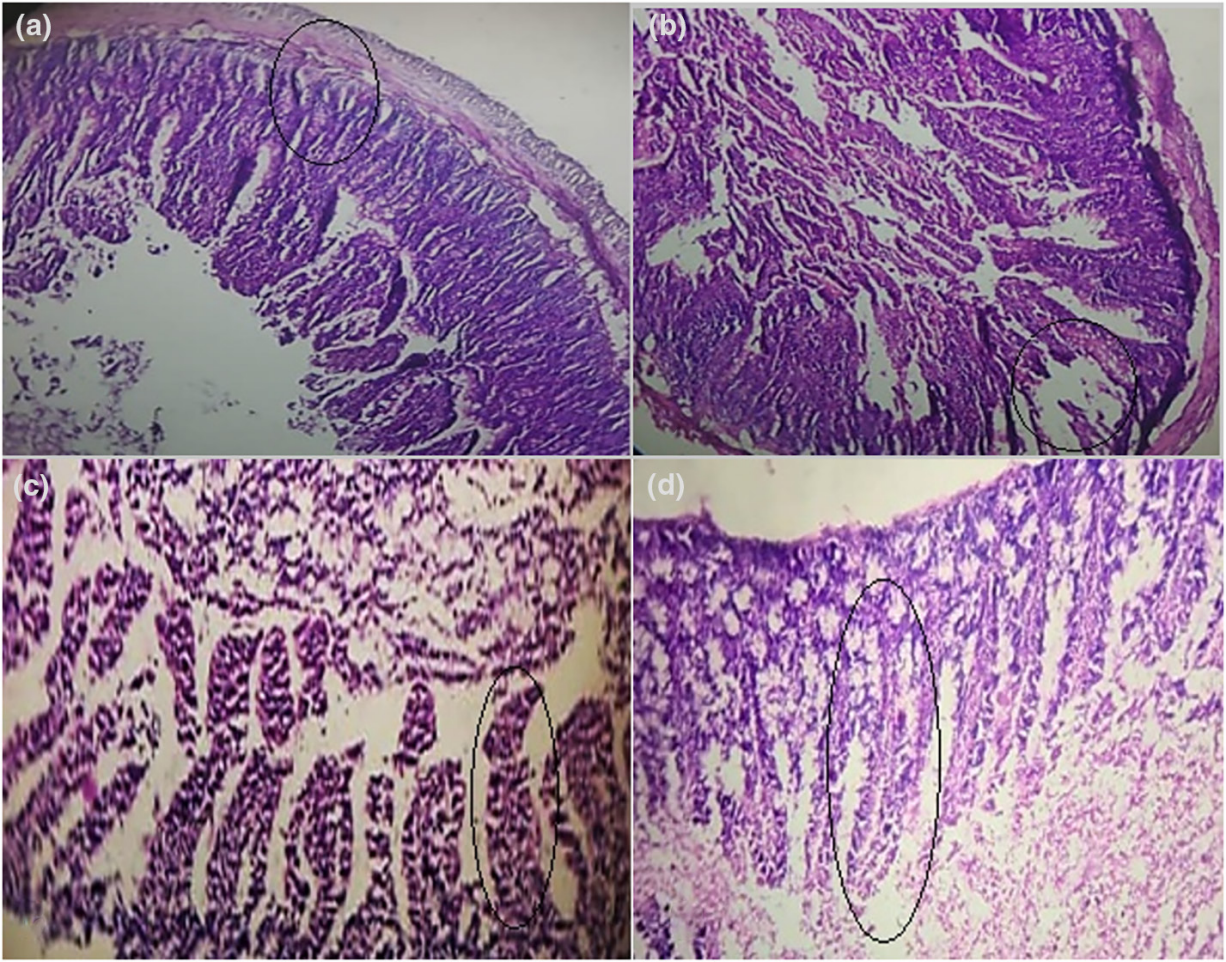
Microscopic observation of the intestine histological section in rats of different groups (×100). (a) Control group; (b) infected group; (c) treated group (*Bacillus safensis*); (d) treated group (*Bacillus safensis* + Jc).

According to the rats that received the probiotic treatment after the infection, the histological sections show a less damaged intestinal structure and the mucosa seems less affected, with a reduction in the rate of inflammation compared with the villi in group II this group presents a partial return of the height of the villi accompanied by the absence of necrosis. The rats that were treated with the combination of *Bacillus safensis* and *Juniperus communis* extract, showed a more effective response to the treatment, where the signs of infection disappeared completely and revealed an almost total return of height of the villi, and the disappearance of all the symptoms that were observed in group II, where we noted a decrease in the severity of inflammation and the absence of necrotic cells, also found a healthy intestinal wall structure with their components compared with the damaged one (Figure 3).

### 3.7 Determination of blood biochemical parameters

#### 3.7.1 Blood count (BCN)

**FIGURE 4:**
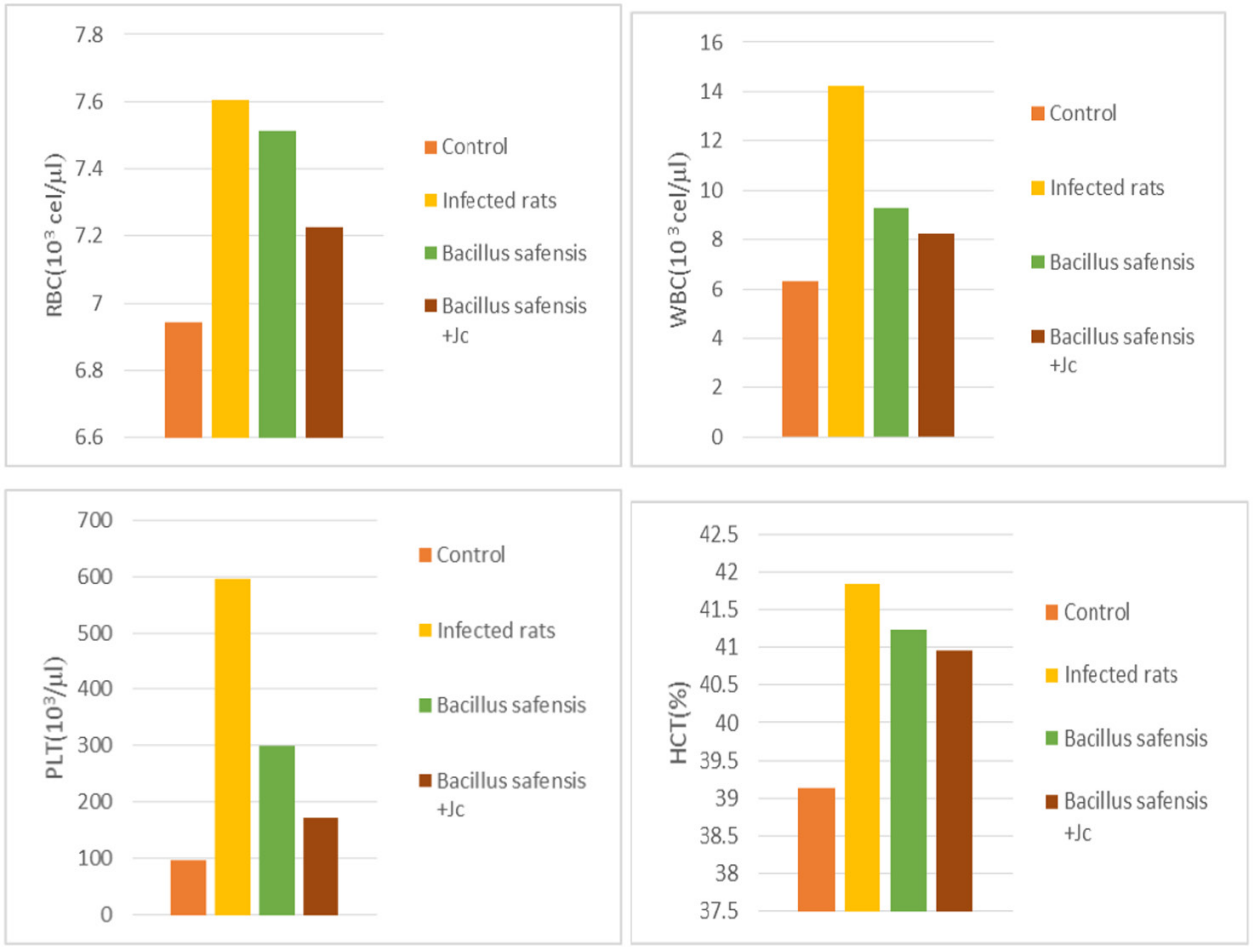
Determination of hematological parameter (BCN) of experimental rats.

From BCN results (Figure 4), it was observed that the target group had an increase in red blood cell levels, hematocrit, platelet count, and white blood cell values compared with the other groups. While it was observed that there was a decrease in these values after treatment with *Bacillus safensis* strains, they returned to almost normal values for the group that received the combination of *Bacillus safensis* and *Juniperus communis* extract.

### 3.8 Inflammatory assessment parameters

#### 3.8.1 Erythrocyte sedimentation rate (ESR)

The ESR results (Table 8) showed that there was a significant increase in the ESR parameter in the infected group compared with others, while it decreased in the treated groups, with more effectiveness recorded in the fourth group.

**TABLE 8:** Determination of inflammatory assessment parameters (CRP, ESR) in experimental rats.

	Group I (control)	Group II (infected)	Group III (*Bacillus safensis* treatment)	Group IV (*Bacillus safensis +* JAE treatment)
ESR (mm/h)	1.53 ± 0.29	5.09 ± 0.32	4.37 ± 0.38	3.96 ± 0.19
CRP (mg/dL)	3.65 ± 0.35	9.93 ± 1.81	6.26 ± 0.71	5.15 ± 0.24

#### 3.8.2 C‐reactive protein (CRP)

The findings demonstrated the presence of severe infections in group II, and treatment with *Bacillus safensis* probiotics in group III reduced the severity of inflammation, also the effectiveness of the mixture (*Bacillus safensis +* JAE treatment) showed a higher anti‐inflammatory effect in group IV compared with group III (Table 8).

## 4 DISCUSSION

HPLC detected seven phenolic components in the aqueous extract of *Juniperus communis*, it was also noted that there was a high content of quercetin because numerous physiological functions in plants, including photosynthesis, pollen development, antioxidant machinery, and seed germination, are made easier by quercetin. The results obtained by Garg (2010), are similar to the results of the qualitative phytochemical analysis. These levels may be influenced by a variety of elements, including storage conditions, extraction techniques, genotype, growth, and maturation circumstances (Benhammou et al., 2007). All isolate strains are Gram‐positive and the form of cells is ovoid or spherical cocci. This result is close to the findings of Hamed and Elattar (2013). The strains are generally catalase (+)‐positive, This shows that the formation of bubbles is a result of bacterial production of the enzyme catalase, which works to eliminate the toxicity of H_2_O_2_ by rupturing the link (Andhikawati & Permana, 2022). All isolates showed better growth at different concentrations of NaCl (2%, 4%, and 6.5%). According to Mhamed et al. (2022), the growth of *L. paracasei* L2 reduced as the NaCl concentration increased (from 2% to 6.5%), and the highest growth rate (61.74%) was recorded at 2% NaCl. Also, Qin et al. (2022), confirmed that probiotics can grow under high‐alkaline and high‐saline conditions. A significant increase in growth is observed for most of the strains at 45°C, this indicates that lactic acid bacteria are able to reproduce at high temperatures. The isolation of *Bacillus safensis* and *Bacillus pumilus* from camel's milk was also reported by Laiche et al. (2019). In this study, we sequenced 16srDNA and compared it with the NCBI database, and we found that the identified strains belong to *Bacillus pumilus* and *Bacillus safensis*. The growth of *Bacillus safensis* and *Bacillus pumilus* increases at pH = 3. Yasmin et al. (2020) researched how well different probiotic bacteria tolerated juices and artificial intestinal gastrics, and they discovered that the isolated strains could live for 2 h at pH levels of 2 and 3. The findings indicate that all strains exhibit better survival rates at bile salt concentrations of 5%. According to several research, probiotics with intestinal origin, including lactobacilli, have evolved a resistance to the bile salts' detergent effect (Begley et al., 2006; Zhang et al., 2023). This may be related to the isolates' capacity to produce an intracellular bile salt hydrolase enzyme (BSH) (Horáčková et al., 2018). The appearance of infection signs in rats is due to the toxic effects of castor oil seeds (*Ricinus communis*, Euphorbiaceae) which contain ricin, and are among the phytotoxins' comparatively well‐researched members. Thus, the first symptom is body weight loss, which is observed around 12 h after the administration of a dose of ricin. It is common to observe diarrhea with inflammation, this is most likely caused by the extensive intestinal lesion (Balint, 1974).

The decrease in inflammation symptoms after administration of *Bacillus safensis* treatment is because this strain of probiotics has beneficial effects on human, mainly at the intestinal level (Heyman & Ménard, 2002). Furthermore, diarrhea can be associated with changes in the intestinal bacterial flora therefore taking doses of *Bacillus safensis* restores their balance (Ko et al., 2011). The symptoms disappeared in group IV which was treated by a combination of *Juniperus communis* and *Bacillus safensis* strains. Evidence suggests that *Juniperus communis* compounds enhance probiotic anti‐inflammatory properties.

Microscopic observation of the histological sections of the rats that received castor oil revealed a destroyed structure of intestinal cells plus the presence of signs of very serious infection represented by inflammation. Because, the majority of bodily tissues and organs are often impacted by ricin. The degree of functional and anatomical alterations resulting from prolonged exposure of a certain organ or tissue to ricin has been seen to vary. Ricine mostly affects the intestines out of all the internal organs since it often enters the body through the intestines and exits through them. This impact likewise primarily affects intestinal cells' smooth endoplasmic reticulum and is linked to a few small alterations in the mitochondria. Additionally, it inhibits the creation of proteins (Balint, 1974; Guo et al., 2024).

The histological sections of group III show a less damaged intestinal structure and the mucosa seems less affected, with a reduction in the rate of inflammation compared with group II, this is due to the fact that probiotics have the potential to strengthen the intestinal barrier through the preservation of tight junctions and stimulation of mucin synthesis. Probiotic‐mediated immunomodulation can also impact the proliferation and development of immune cells (like T cells) or epithelial cells (Hu et al., 2022). It can also happen through the mediation of cytokine release through signaling pathways like NFκB and MAPKs (Hemarajata & Versalovic, 2013). The rats treated with the combination of *Bacillus safensis* and *Juniperus communis* extract had a more effective response to the treatment than group III, where the signs of infection disappeared completely. This is because the active components of the extracts balanced the intestinal flora, which, during intestinal transit, released the active metabolites that may pass through the intestinal barrier and have anti‐inflammatory properties. Under normal circumstances, this would strengthen the intestinal microenvironment's inhibitory tone and aid in the downregulation of inflammation under pathological circumstances (Menard et al., 2004).

It was observed that the infected group had an increase in red blood cell levels, hematocrit, platelet count, and white blood cell values similar to those in infectious diseases. The decrease in these hematological indices in group III is due to the fact that probiotics improve blood biochemical parameters (Biswas et al., 2022). While in group IV, It was found that *Juniperus communis* enhanced *Bacillus safensis* properties, including the beneficial effects on the hematology of rats.

The high values of CRP and ESR tests reported in group II indicate the occurrence of severe intestinal infections, this is due to the toxic effects of the ricin compound in castor oil. The decrease in CRP and ESR test values in group III rats indicates the disappearance of inflammatory symptoms, it has been demonstrated that lactobacilli, bifidobacteria, or probiotic combinations can treat digestive disorders in experimental animals (Madsen et al., 1999, 2001) as well as inflammatory bowel diseases in humans (Borruel et al., 2002; Gionchetti et al., 2000; Gupta et al., 2000). Early *Bifidobacterium infantis* treatment prevented the development of necrotizing enterocolitis in newborn rats with stress‐induced enterocolitis.

The effectiveness of *Bacillus safensis* and *Juniperus communis* mixture that was administrated to group IV in eliminating inflammatory indicators was more effective compared with group III, This was proven through Palhares Campolina et al. (2021) studies on the effect of the same plant species on modifying intestinal flora and thus reducing digestive diseases due to the active components of the extracts. Then the balance of intestinal flora increases the production and secretion of interleukin 10 in macrophages and T cells originating from the inflamed colon (Pathmakanthan et al., 2004); Probiotic strains generate compounds that can pass the intestinal barrier and have an anti‐tumor necrosis factor‐alpha effect (Menard et al., 2004).

## 5 CONCLUSION

This research's goal was to examine how *Juniper communis* plant extract affects on probiotic properties of *Bacillus safensis* isolated from camel's milk in the region of El Oued (Algeria). The extracts are high in quercetin, according to the chromatographic profiles of the phenolic components. The quantitative analysis of the extract from *Juniperus communis* showed that the aqueous extract is rich in polyphenols (103.80 ± 0.30 mg GAE/g E), and contains a significant amount of flavonoids (15.85 ± 0.80 mg QE/g E). Probiotic strains are isolated from camel milk, it is rich in *Bacillus safensis*, and *Bacillus pumilus*. The results of this study showed that *Juniperus communis* which contains a significant amount of polyphenols and flavonoids can play a major role in the activity of probiotic bacteria such as *Bacillus safensis*. This demonstrates that it has a favorable impact on the digestive system.

## AUTHOR CONTRIBUTIONS

**Ikram Layadi:** Resources (equal); software (equal); supervision (equal). **Ammar Touhami Laiche:** Resources (equal); software (equal). **Mohammed Laid Tlili:** Resources (equal); software (equal). **Mohammed Messaoudi:** Conceptualization (equal); validation (equal); writing – original draft (equal). **Djilani Ghemam Amara:** Conceptualization (equal); validation (equal); writing – original draft (equal). **Maha Mezghani‐Khemakhem:** Resources (equal); software (equal). **Chahnez Naccache:** Resources (equal); software (equal). **Barbara Sawicka:** Conceptualization (equal); validation (equal); writing – original draft (equal). **Maria Atanassova:** Resources (equal); validation (equal). **Wafa Zahnit:** Resources (equal); software (equal); validation (equal); writing – review and editing (equal). **Sheikh F. Ahmad:** Resources (equal); software (equal); supervision (equal); validation (equal); visualization (equal); writing – review and editing (equal).

## FUNDING INFORMATION

This research was funded by King Saud University, Riyadh, Saudi Arabia, Project Number (RSPD2024R709).

## CONFLICT OF INTEREST STATEMENT

The authors declare no conflicts of interest.

## Data Availability

The datasets used and/or analyzed during the current study are available from the corresponding author on reasonable request.
